# ﻿Functional traits of ancestral caddisfly (Trichoptera) larvae and pupae

**DOI:** 10.3897/zookeys.1263.148069

**Published:** 2025-12-10

**Authors:** Xinyu Ge, John C. Morse

**Affiliations:** 1 Tianjin Key Laboratory of Conservation and Utilization of Animal Diversity, College of Life Sciences, Tianjin Normal University, Tianjin, 300387, China Tianjin Normal University Tianjin China; 2 Department of Plant and Environmental Sciences, Clemson University, Clemson, South Carolina, 29634-0310, USA Clemson University Clemson United States of America

**Keywords:** Bryophytes, case, cocoon, pupa, retreat, silk

## Abstract

Recent phylogenomic studies have concluded that the ancestor of order Trichoptera and suborder Integripalpia probably had a larva that was “free living,” without a portable case or fixed retreat. Phylogenies inferred from those investigations regarding hypotheses for other probable functional traits of larvae and pupae of the Trichoptera ancestor and its immediate descendants were considered, especially with reference to the extant amphiesmenopteran sister lineage Lepidoptera. To test our hypotheses an Ancestral Character State Reconstruction by Parsimony Analysis was performed to explore functional traits for five habitat and behavioral traits. Like the larva of Micropterigidae, the basal lineage of Lepidoptera, the ancestral caddisfly larva was not only “free living” but also was a shredding herbivore of bryophytes. Like that larva, it may have been often submerged, perhaps as a semi-aquatic sprawler in madicolous or hygropetric habitats, but it could also have been a clinger in lotic-erosional habitats. Also, the characteristics of the pupal cocoon are not clear; it may have been closed and permeable like that of Micropterigidae, or it was closed and semipermeable like that of Hydroptilidae, or it was open in a long-dome shelter like that of the Annulipalpia ancestor.

## ﻿Introduction

Ecologically, larvae of caddisflies (the insect order Trichoptera) are more diverse than those of almost any other order of freshwater macroinvertebrates and are similar in ecological diversity to larvae of freshwater flies, or Diptera (Merritt et al. 2019). Larvae of the megadiverse moths, the trichopteran sister order Lepidoptera according to Misof et al. (2014), are mostly terrestrial herbivores. Therefore, a long-standing question has been how caddisfly larvae made the very dramatic evolutionary transition from a purely terrestrial habitat to an aquatic habitat. What did the ancestral caddisfly larvae look like? What did they eat? In what habitat did they live and what behaviors allowed them to live there successfully? In what type of enclosure did they pupate? With the less-well-developed phylogenetic evidence known at the time of their studies, Milne and Milne (1939) thought ancestral Trichoptera larvae inhabited ponds and slow streams and Ross (1956) concluded “that the primitive caddisflies were cool-adapted forms living in running streams.” Either of these ideas would have required multiple morphological, physiological, and behavioral adaptations to have evolved simultaneously, a seemingly improbable scenario. With regard to pupae, Wiggins (2004) inferred that in the ground plan of Trichoptera “pupation [occurred] at [the] end of [the] final larval instar in [a] domed enclosure, open to moving water” and beneath this dome they constructed a “closed, semipermeable cocoon free from [the] wall of [the] dome.”

What were the functional traits of ancestral caddisfly larvae and pupa and what were the structural and functional evolutionary characteristics that enabled their successful invasion of resource-rich freshwater habitats? Recent revisions of moth phylogeny provided by Mitter et al. (2017) and Kawahara et al. (2019) placed Micropterigidae and Agathaphagidae as the earliest successive lineages of Lepidoptera. Recent revisions of caddisfly phylogeny also were provided by Thomas et al. (2020), Frandsen et al. (2024), and Ge et al. (2024a), placing Hydropsychoidea as the basal lineage for all other Annulipalpia and Hydroptiloidea as the basal lineage for all other Integripalpia. These recent authors also determined the time of origin for those orders as Late Carboniferous (~300 MA) for Lepidoptera (Kawahara et al. 2019) and Permian (~275 MA by Thomas et al. 2020; ~295 MA by Frandsen et al. 2024; 281.16–302.52 MA by Ge et al. 2024a) for Trichoptera. Through the investigations of many colleagues summarized in publications such as those by Graf et al. (2008) and Merritt et al. (2019), we also have a much improved understanding of the functional traits of the basal lineages of Lepidoptera and Trichoptera and their included families.

Based on those resources, we presented our hypothesis at the 18^th^ International Symposium on Trichoptera (Quito, Ecuador, 1–5 July 2024) that the larva of the ancestral caddisfly was not only free-living (Wiggins 2004; Frandsen et al. 2024; Ge et al. 2024a), but also a shredding herbivore feeding on bryophytes in wet and sometimes submerged or hygropetric environments. Reviewers for the manuscript submitted for the Proceedings of the symposium encouraged us to conduct a parsimony analysis to test this hypothesis, an analysis which we agreed to undertake.

Currently, the superorder Amphiesmenoptera Kiriakoff, 1948, consists of four orders and ~211 extant and fossil (†) families (Morse 2025; van Nieukerken et al. 2011):

† Order Tarachoptera Mey, Wichard, Müller & Wang, 2017a;

† Family Tarachocelidae Mey, Wichard, Ross & Ross, 2017b;

† Order Protomeropina Tillyard, 1926;

† Family Cladochoristidae Riek, 1953;

† Family Kalophryganeidae Haupt, 1956;

† Family Karaungiridae Novokshonov & Sukatsheva, 1997;

† Family Microptysmatidae Martynova, 1958;

† Family Protomeropidae Tillyard, 1926;

† Family Terminoptysmatidae Melnitsky & Ivanov, 2020 (in Kopylov et al. 2020);

† Family Uraloptysmatidae Ivanov, 1992;

Order Trichoptera ~51 extant fam., 14 fossil fam.;

Order Lepidoptera ~134 extant fam., 4 fossil fam.

Among these four orders, Mey et al. (2017b) and Mey and Wichard (2023) considered Lepidoptera and Trichoptera (and their respective stem groups and sister groups) as sister lineages. For Tarachoptera and Protomeropina, DNA sequences are unavailable for phylogenetic analysis, and nothing is known about the structures, functions, and habitats of their immature stages.

### ﻿Order Lepidoptera

Among the families of Lepidoptera, Mitter et al. (2017) considered that Micropterigidae is probably the basal lineage but said that “the question is not fully settled.” If correct, however, then they concluded that the gymnosperm-seed-feeding family Agathiphagidae is the next successive family in the order and the sister family of all other Lepidoptera, the clade Angiospermavora. The phylogenomic study of Kawahara et al. (2019) supported these relationships.

Larvae of micropterigids feed on foliose liverworts, mosses, or detritus and live in or on the ground (Mitter et al. 2017) without constructed cases or shelters (i.e., are “free living”). Davis and Landry (2012) provided further details for two of the three species of the North American micropterigid genus *Epimartyria*: *E. auricrinella* Walsingham, 1898, and *E. pardella* (Walsingham 1880). *Epimartyria auricrinella* larvae feed on liverworts (*Bazzania trilobata*) in shaded, wet locations (swampy woods, boggy ditches, creek margins), “which probably differed little from those of ancestral ‘Amphiesmenoptera’ ” (Kristensen 1997), and that can be flooded periodically or seasonally. They are peripneustic with spiracles on abdominal segments I–VIII and a plastron supporting a thin layer of air on each side around the spiracles and over the posterior pronotum, permitting survival when submerged for at least short periods. Eggs of *E. pardella* are laid on the ventral surfaces of liverwort (*Conocephalum conicum*) and larvae also spend days on the undersides of liverwort thalli; “pupation occurs within a thin-walled, tightly woven brown cocoon close to the ground and attached to vegetation with strands of coarse silk” (Davis and Landry 2012); no other pupal shelter was mentioned. For basal lineages of Lepidoptera, silk production is confined to construction of cocoons by last-instar larvae (Kristensen 1997).

### ﻿Order Trichoptera

In multigene and phylogenomic studies, Thomas et al. (2020), Frandsen et al. (2024), and Ge et al. (2024a) all concluded that Trichoptera and suborders Annulipalpia and Integripalpia are each monophyletic. Furthermore, they concluded that the relationships within Annulipalpia were (Hydropsychoidea (Philopotamoidea + Psychomyioidea). Within Integripalpia, those three studies also concluded that the basal lineage is a clade of Hydroptiloidea sister families Ptilocolepidae and Hydroptilidae (or Hydroptilidae only, Ptilocolepidae having not been studied by Ge et al. 2024a). Frandsen et al. (2024) and Ge et al. (2024a) also inferred that the remaining families of Integripalpia are a clade with relationships (Phryganides (Glossosomatidae + Rhyacophiloidea)), for which subterorder Phryganides Latreille, 1805, consists of all families with tube-case-making larvae.

In modern Annulipalpia, campodeiform larvae usually construct silken retreats in all instars to provide physical protection from predators, usually attached to stable substrate surfaces in lotic waterways. These retreats may be augmented with specialized filter nets to one side of the upstream end (Hydropsychoidea and Stenopsychidae) or much of the inner surface of the retreat itself may filter food particles (Philopotamidae and some Psychomyioidea). Some Psychomyioidea larvae use their retreats as snares resembling spider webs for capturing drifting prey, as lairs from which to pounce on unsuspecting prey or to graze on periphyton, as reinforcement for subterranean tunnels with filter nets (Wiggins 2004), or as substrate for culturing algae (Ings et al. 2010, 2017).

In modern Integripalpia, Hydroptiloidea (Hydroptilidae + Ptilocolepidae) larvae undergo hypermetamorphosis, with the first four instars having a typical campodeiform shape (Nielsen 1948) and then, in the fifth and final instar, becoming hypergastric, developing a much-enlarged abdomen. Notably, the early-instar campodeiform larvae are “free-living,” with only the last instar constructing a sagitally compressed or transversely depressed case of silk or of silk and plant or mineral substrate sealed laterally on two longitudinal seams; this case is usually portable but sometimes fixed to substrate (Wiggins 2004). In particular, much like at least some lepidopteran micropterigid larvae, Ptilocolepidae larvae shred living mosses and liverworts in hygropetric or madicolous, damp streamside habitats, typically above the water line. Furthermore, the final instar, with its much-enlarged abdomen, constructs a portable, transversely depressed case of pieces of liverwort (Thienemann 1904; Ito and Higler 1993; González et al. 2000; Wiggins 2004; Ito et al. 2014). The final instar of a hydroptiloid larva pupates in a silken cocoon that is either semipermeable (Hydroptilidae) or permeable (Ptilocolepidae) (Wiggins 2004).

In all larval instars, Glossosomatidae larvae construct so-called “tortoise” cases of mineral pieces from which they graze periphyton off the upper surfaces of rocks in rapidly flowing water. Each glossosomatid case consists of a dome-like dorsal covering attached laterally to a flat, transverse, ventral strap or “plastron” that leaves identical, interchangeable openings for the head and legs at one end and the tip of the abdomen at the other; prior to pupation, the fifth (last) larval instar removes the ventral strap, seals the remaining dome to the rock, and spins a semipermeable cocoon beneath it (Wiggins 2004). Campodeiform Rhyacophiloidea (Hydrobiosidae + Rhyacophilidae) larvae build no cases until just prior to pupation but roam freely over the substrate; they feed generally as predators, but sometimes scrape periphyton, collect fine particulate organic matter, or shred living plant tissue (Cummins et al. 2019). The dome-like shelter built by the mature larva resembles that of Glossosomatidae, under which the rhyacophiloid larva also spins a semipermeable cocoon before pupating (Wiggins 2004).

Tube-case making Phryganides larvae typically construct their cases in all instars. The earliest lineages of infraorder Plenitentoria Weaver, 1984, do so with angiosperm pieces and those of infraorder Brevitentoria Weaver, 1984, make cases with mineral materials. When preparing for pupation, they usually attach their larval cases to stable substrate and close the ends with tough silken mesh sieves to deter predators while allowing flow of oxygenated water (Wiggins 2004).

With apparent radiation of so much structural and functional diversity in freshwater ecosystems, we were curious to learn how the ancestral caddisfly larva invaded its aqueous habitat and what evolutionary adaptations allowed it to succeed in that environment.

## ﻿Methods

For each of the families for which phylogenetic relationships were inferred by Ge et al. (2024a) and with the addition of Ptilocolepidae (from Frandsen et al. 2024), and the basal lineages of Lepidoptera (Mitter et al. 2017; Kawahara et al. 2019), we determined from the most recent available studies the basal lineages of Lepidoptera and families of Trichoptera and the traits of those lineages (Tables 1–3). From among these, we selected five trait categories of the larvae, including the Case-Retreat, Food (= Feeding or Trophic Relationships), Habitat, Cocoon Construction, and Habit using terminology mainly from Cummins et al. (2019). For each trait category, the known trait of the ancestral lineage of each family was encoded for analysis as in Tables 1–3.

**Table 1.** Ancestral traits for case-retreat and food-feeding-trophic relationships of larvae of basal taxa of Lepidoptera (Kawahara et al. 2019) and the Trichoptera families for which phylogenetic relationships were inferred by Ge et al. (2024a), with Ptilocolepidae from Frandsen et al. (2024), showing color codes for inferred ancestral nodes in Fig. 1.

Taxon	Case-Retreat^1^ (Trait 1)	Codes for Trait 1	Food-Feeding-Trophic Relationships^2^ (Trait 2)	Codes for Trait 2	Reference(s) for Inferred Basal Lineage(s) of Terminal Taxa	Reference(s) for Traits of Basal Lineage(s)
Angiospermavora	None (free living, fl).	0 (white)	Shredding herbivores (angiosperms) (shh).	0 (white)	Mitter et al. 2017, Kawahara et al. 2019.	Stehr 1987 (Angiospermavora families).
Heterobathmiidae	None (free living, fl).	0 (white)	Shredding herbivores (*Nothofagus*, angiosperms) (shh).	0 (white)	Mitter et al. 2017, Kawahara et al. 2019.	Kristensen and Nielsen 1983.
Agathiphagidae	None (free living, fl).	0 (white)	Shredding herbivores (*Kauri* seeds, gymnosperms) (shh).	0 (white)	Mitter et al. 2017, Kawahara et al. 2019.	Dumbleton 1952.
Micropterigidae	None (free living, fl).	0 (white)	Shredding herbivores (bryophytes) (shh).	5 (black)	Mitter et al. 2017, Kawahara et al. 2019.	Davis and Landry 2012.
Ptilocolepidae	None (free living early instars) →portable rigid purse case (last instar; fl, pc).	0 (white)	Shredding herbivores (bryophytes) (shh).	5 (black)	*Palaeagapetus* and *Ptilocolepus* (the only genera).	Ito et al. 2014; Waringer and Graf 2002.
Hydroptilidae	None (free living early instars) →portable rigid purse case (last instar; fl, pc).	0 (white)	Grazers (gra).	1 (brown)	*Stactobia* (Marshall 1979).	Graf et al. 2008; Waringer and Graf 2011.
Glossosomatidae	Portable rigid tortoise case (ps).	4 (black)	Grazers (gra).	1 (brown)	*Anagapetus* and *Glossosoma* (Robertson and Holzenthal 2013).	Wiggins 1996; Cummins et al. 2019.
Hydrobiosidae	None (free living, fl).	0 (white)	Predators (pre).	2 (green)	*Apsilochorema* (Ward et al. 2004).	Holzenthal et al. 2007.
Rhyacophilidae	None (free living, fl).	0 (white)	Predators (pre).	2 (green)	*Fansipangana* (larva unknown) and *Rhyacophila* (McLaughlin et al. 2019).	Graf et al. 2008.
Phryganopsychidae	Limp plant tube case (pl).	1 (brown)	Shredding detritivores (shd).	3 (yellow)	*Phryganopsyche* (only genus).	Wiggins 2004.
Pisuliidae	Rigid plant tube case (plant, pl).	1 (brown)	Shredding detritivores (shd).	3 (yellow)	*Pisulia* and *Silvatares* (Stoltz 1989).	Stoltz 1989.
Phryganeidae	Rigid mineral and plant tube case (mn and pl).	1 (brown)	Predators (pre).	2 (green)	*Yphria* (Wiggins 1998).	Wiggins 1998.
Brachycentridae	Rigid plant tube case (pl).	1 (brown)	Shredding herbivores (shh).	0 (white)	*Eobrachycentrus* (Flint 1984).	Wiggins 1965, 1996.
Lepidostomatidae	Rigid tube case (mineral or plant, mn or pl).	1 (brown)	Shredding detritivores (shd).	3 (yellow)	Basal lineages of Lepidostomatinae and Theliopsychinae have not been inferred.	Wiggins 2004; Cummins et al. 2019.
Uenoidae	Rigid silk tube case (mineral, mn; si).	2 (green)	Grazers and gatherers (gra, gat).	1 (brown)	*Sericostriata* (Wiggins et al. 1985).	Wiggins et al. 1985.
Goeridae	Rigid mineral tube case (mn).	2 (green)	Grazers (gra).	1 (brown)	Basal lineages of Goerinae, Larcasinae, and Lepaniinae have not been inferred. Traits are almost universal in the family.	Wiggins 1996, 2004.
Apataniidae	Rigid mineral tube case (mn).	2 (green)	Grazers (shd).	1 (brown)	*Apataniana* (Ge et al. 2024b).	Waringer and Malicky 2016.
Limnephilidae	Rigid mineral tube case (mn; plant, pl).	2 (green)	Shredding detritivores; gatherers (shd, gat).	? (stripe)	*Ecclisomyia*, *Philocasca* (Vshivkova et al. 2007).	Wiggins 2004; Cummins et al. 2019.
Limnocentropodidae	Rigid mineral tube case (mn).	2 (green)	Predators (pre).	2 (green)	*Limnocentropus* (Wiggins 2004).	Wiggins 2004.
Odontoceridae	Rigid mineral tube case (mn).	2 (green)	Predators (pre).	2 (green)	Two subfamilies: monotypical Pseudogoerinae and polytypical Odontocerinae, but basal lineages of Odontocerinae have not been inferred. (Wallace and Ross 1971).	Wallace and Ross 1971 (*Pseudogoera*); Wiggins 1996; Holzenthal et al. 2007 (Odontocerinae).
Helicopsychidae	Rigid mineral tube case (mn).	2 (green)	Grazers (gra).	1 (brown)	*Rakiura* (Johanson 1998; Johanson et al. 2017).	Traits of *Rakiura* not described, apparently as for *Helicopsyche* (Johanson 1998).
Sericostomatidae	Rigid mineral tube case (mn).	2 (green)	Shredding herbivores, (shh).	0 (white)	[Larvae of basal *Cheimacheramus*, *Rhoizema*, *Petroplax*, and *Notidobiella* unknown] *Gumaga* (Johanson et al. 2017).	Resh et al. 1997.
Calamoceratidae	Rigid plant tube case (pl).	1 (brown)	Shredding detritivores (shd).	3 (yellow)	Two subfamilies: Monotypical Anisocentropinae and polytypical Calamoceratinae, but basal lineages of Calamoceratinae have not been inferred (Prather 2002).	Wallace and Sherberger 1970 (*Anisocentropus*).
Molannidae	Rigid mineral tube case (mn).	2 (green)	Collecting gatherers, predators (gat, pre).	? (stripe)	*Molanna* and *Mollanodes* (the only genera).	Graf et al. 2008; Cummins et al. 2019.
Leptoceridae	Rigid mineral or plant or silk tube case (mn or pl or si).	? (stripe)	Collecting gatherers, shredding herbivores, grazers, predators (gat, shh, gra, pre).	? (stripe)	*Grumichella* and *Leptorussa* (Malm and Johanson 2011); *Russobex* (St Clair 1988).	Case-retreat and trophic relations of *Grumichella*, *Leptorussa*, and *Russobex* unknown.
Hydropsychidae	Fixed silk+substrate retreat with filter net (rt, fn).	3 (yellow)	Collecting filterers (pff).	4 (red)	Arctopsychinae: *Arctopsyche*, *Parapsyche* (Geraci et al. 2005).	Cummins et al. 2019; Graf et al. 2008.
Stenopsychidae	Fixed silk+substrate retreat with filter net (rt, fn).	3 (yellow)	Collecting filterers (pff).	4 (red)	*Pseudostenopsyche* and *Stenopsychodes* larvae unknown; *Stenopsyche* is the only other genus in the family.	Tanida 2002.
Philopotamidae	Fixed silk retreat used as filter net (rt, fn).	3 (yellow)	Collecting filterers (pff).	4 (red)	Larvae of basal subfamily Rossodinae (*Rossodes*) are unknown (Gibon 2013; Blahnik 2005); larvae of both remaining subfamilies ecologically similar.	Graf et al. 2008; Cummins et al. 2019.
Dipseudopsidae	Fixed silk+substrate retreat in buried tube (tm, fn).	3 (yellow)	Collecting filterers (pff).	4 (red)	Subfamilies Dipseudopsinae and Hyalopsychinae (the only subfamilies) (Ross and Gibbs 1973).	Ulmer 1957; Marlier 1962; Gibbs 1968 (Dipseudopsinae: *Dipseudopsis*, *Protodipseudopsis*); Wallace et al. 1976 (Hyalopsychinae: *Phylocentropus*).
Pseudoneureclipsidae	Short fixed silk retreat in sand tube on ventral surface of rock (tm, rt).	3 (yellow)	Collecting filterers (pff) possibly using vortices (“eddying currents”--Gibbs 1973).	4 (red)	*Pseudoneureclipsis* and *Antillopsyche* (the only genera).	Gibbs 1973; Tachet et al. 2001 (*Pseudoneureclipsis*).
Xiphocentronidae	Long, fixed meandering, tubular silk+substrate retreat on substrate surface often extending above water line (tm, rt).	3 (yellow)	Collecting gatherers (gat).	1 (brown)	Proxiphocentroninae: *Proxiphocentron*, larva unknown (Schmid 1982); Xiphocentroninae: *Drepanocentron* (Vilarino et al. 2022).	Genco et al. 2020 (*Drepanocentron*).
Psychomyiidae	Long, fixed, meandering, tubular silk+substrate retreat on substrate surface (tm, rt).	3 (yellow)	Grazers (gra).	1 (brown)	(Psychomyiinae: *Metalype*), (Tinodinae: *Lype*) (Li and Morse 1997).	Edington and Hildrew 1995 (*Metalype*, *Lype*).
Ecnomidae	Long, fixed, meandering, tubular silk+substrate retreat on substrate surface (tm, rt).	3 (yellow)	Predators (pre).	2 (green)	*Daternomina*, *Parecnomina* (Johanson and Espeland 2010).	Cartwright 1997 (*Daternomina*); Gibbs 1973 (*Parecnomina*).
Polycentropodidae	Fixed silk retreat with filtering function (rt, fn).	3 (yellow)	Collecting filterers, predators (pff, pre).	2 (green)	*Neureclipsis* (Johanson et al. 2012).	Edington and Hildrew 1995; Inaba et al. 2014; Wiggins 1996 (*Neureclipsis*).

^1^fl = free-living; fn = filternet with retreat; mn = tubular mineral case; pc = purse case; pl = tubular plant case; ps = plastron-strap “tortoise” case; rt = silk retreat; si = tubular silk case; tm = tubular retreat with mineral particles. ^2^gat = gatherers-scrapers of sedimented fine particulate organic matter (FPOM = organic particles <10μ3); gra = grazers-scrapers of endolithic and epilithic algal tissues, biofilm, some FPOM, some tissues of living plants; pff = passive filter feeders of suspended FPOM and coarse particulate organic matter (CPOM = mainly fallen, entrained, and microbially conditioned leaves and other dead organic tissue >10μ3) and meiofauna and microfauna from moving water using nets or leg hairs and specialized mouthparts; pie = piercers of filamentous algae; pre = predators; shd = shredding detritivores of CPOM; shh = shredding herbivores of living plant tissue.

**Table 2.** Ancestral traits for habitat and cocoon of larvae and pupae of basal taxa of Lepidoptera (Kawahara et al. 2019) and the Trichoptera families for which phylogenetic relationships were inferred by Ge et al. (2024a), with Ptilocolepidae from Frandsen et al. (2024), showing color codes for inferred ancestral nodes in Fig. 2.

Taxon	Habitat (Trait 3)	Codes for Trait 3	Cocoon^1^ (Trait 4)	Codes for Trait 4	Reference(s) for Inferred Basal Lineage(s) of Terminal Taxa	Reference(s) for Traits of Basal Lineage(s)
Angiospermavora	Terrestrial.	3 (brown)	New, closed, permeable, free cocoon.	0 (white)	Mitter et al. 2017, Kawahara et al. 2019.	Stehr 1987 (Angiospermavora families).
Heterobathmiidae	Terrestrial.	3 (brown)	New, closed, permeable, free cocoon.	0 (white)	Mitter et al. 2017, Kawahara et al. 2019.	Kristensen and Nielsen 1983.
Agathiphagidae	Terrestrial.	3 (brown)	No cocoon.	4 (stripe)	Mitter et al. 2017, Kawahara et al. 2019.	Dumbleton 1952.
Micropterigidae	Wet soil-hygropetric.	0 (white)	New, closed, permeable, free cocoon.	0 (white)	Mitter et al. 2017, Kawahara et al. 2019.	Davis and Landry 2012.
Ptilocolepidae	Madicolous-hygropetric.	0 (white)	Closed, permeable cocoon in larval purse case.	0 (white)	*Palaeagapetus* and *Ptilocolepus* (the only genera).	Ito et al. 2014; Waringer and Graf 2002.
Hydroptilidae	Madicolous-hygropetric, lotic, lentic.	0 (white)	Closed, semipermeable cocoon in larval purse case.	3 (black)	*Stactobia* (Marshall 1979).	Graf et al. 2008; Waringer and Graf 2011.
Glossosomatidae	Lotic-erosional.	1 (green)	Closed, semipermeable cocoon in larval dome case.	3 (black)	*Anagapetus* and *Glossosoma* (Robertson and Holzenthal 2013).	Wiggins 1996; Cummins et al. 2019.
Hydrobiosidae	Lotic-erosional.	1 (green)	Closed, semipermeable cocoon in new dome case.	3 (black)	*Apsilochorema* (Ward et al. 2004).	Holzenthal et al. 2007.
Rhyacophilidae	Lotic-erosional.	1 (green)	Closed, semipermeable cocoon in new dome case.	3 (black)	*Fansipangana* (larva unknown) and *Rhyacophila* (McLaughlin et al. 2019).	Graf et al. 2008.
Phryganopsychidae	Lotic-depositional.	2 (black)	Closed, permeable cocoon in new tube case.	0 (white)	*Phryganopsyche* (only genus).	Wiggins 2004.
Pisuliidae	Lotic-depositiional (*Silvatares*), hygropetric (*Pisulia*).	2 (black)	Open, in larval silk-lined tube case.	1 (brown)	*Pisulia* and *Silvatares* (Stoltz 1989).	Stoltz 1989.
Phryganeidae	Lotic-depositional.	2 (black)	Open, in new silk-lined tube case.	1 (brown)	*Yphria* (Wiggins 1998).	Wiggins 1998.
Brachycentridae	Lotic-erosional.	1 (green)	Open, in larval silk-lined tube case.	1 (brown)	*Eobrachycentrus* (Flint 1984).	[Bibr B88][Bibr B89].
Lepidostomatidae	Lotic-erosional or depositional.	1 (green)	Open, in larval silk-lined tube case.	1 (brown)	[Basal lineages of Lepidostomatinae and Theliopsychinae have not been inferred.]	Wiggins 2004; Cummins et al. 2019.
Uenoidae	Lotic-erosional.	1 (green)	Open, in larval silk-lined tube case.	1 (brown)	*Sericostriata* (Wiggins et al. 1985).	Wiggins et al. 1985.
Goeridae	Lotic-erosional.	1 (green)	Open, in larval silk-lined tube case.	1 (brown)	[Basal lineages of Goerinae, Larcasinae, and Lepaniinae have not been inferred. Traits in bold are almost universal in the family.]	Wiggins 1996, 2004
Apataniidae	Lotic-erosional.	1 (green)	Open, in larval silk-lined tube case.	1 (brown)	*Apataniana* (Ge et al. 2024b).	Waringer and Malicky 2016.
Limnephilidae	Lotic-erosional (small mountain streams), lentic.	1 (green)	Open, in new silk-lined tube case.	1 (brown)	*Ecclisomyia*, *Philocasca* (Vshivkova et al. 2007).	Wiggins 2004; Cummins et al. 2019.
Limnocentropodidae	Lotic-erosional.	1 (green)	Open, in larval silk-lined tube case.	1 (brown)	*Limnocentropus* (Wiggins 2004).	Wiggins 2004.
Odontoceridae	Lotic-depositional.	2 (black)	Open, in larval silk-lined tube case.	1 (brown)	Two subfamilies: monotypical Pseudogoerinae and polytypical Odontocerinae, but basal lineages of Odontocerinae have not been inferred. (Wallace and Ross 1971).	Wallace and Ross 1971 (*Pseudogoera*); Wiggins 1996; Holzenthal et al. 2007 (Odontocerinae).
Helicopsychidae	Lotic-erosional.	1 (green)	Open, in larval silk-lined tube case.	1 (brown)	*Rakiura* (Johanson 1998; Johanson et al. 2017).	Traits of *Rakiura* not described, apparently as for *Helicopsyche* (Johanson 1998).
Sericostomatidae	Lotic-depositional.	2 (black)	Open, in larval silk-lined tube case.	1 (brown)	Larvae of basal *Cheimacheramus*, *Rhoizema*, *Petroplax*, and *Notidobiella* unknown; *Gumaga* (Johanson et al. 2017).	Resh et al. 1997.
Calamoceratidae	Lotic-depositional.	2 (black)	Open, in larval silk-lined tube case.	1 (brown)	Two subfamilies: monotypical Anisocentropinae and polytypical Calamoceratinae, but basal lineages of Calamoceratinae have not been inferred (Prather 2002).	Wallace and Sherberger 1970 (*Anisocentropus*).
Molannidae	Lentic-erosional.	1 (green)	Open, in larval silk-lined tube case.	1 (brown)	*Molanna* and *Mollanodes* (the only genera).	Graf et al. 2008; Cummins et al. 2019.
Leptoceridae	Lotic-erosional (fast, cool, mountain streams).	1 (green)	Open, in larval silk-lined tube case.	1 (brown)	*Grumichella* and *Leptorussa* (Malm and Johanson 2011); *Russobex* (StClair 1988).	Habitat of *Leptorussa* unknown; habitat of *Grumichella* and *Russobex* mentioned by Calor et al. (2016) and St Clair (1988).
Hydropsychidae	Lotic-erosional.	1 (green)	Open cocoon in new, long-dome shelter.	2 (green)	Arctopsychinae: *Arctopsyche*, *Parapsyche* (Geraci et al. 2005).	Cummins et al. 2019; Graf et al. 2008.
Stenopsychidae	Lotic-erosional.	1 (green)	Open cocoon in new, long-dome shelter.	2 (green)	*Pseudostenopsyche* and *Stenopsychodes* larvae unknown; *Stenopsyche* is the only other genus in the family.	Tanida 2002.
Philopotamidae	Lotic-erosional (Cummins et al. 2019), lotic-depositional (Graf et al. 2008).	1 (green)	Open or closed cocoon in new, long-dome shelter.	2 (green)	Larvae of basal subfamily Rossodinae (*Rossodes*) are unknown--Gibon 2013; Blahnik 2005; larvae of both remaining subfamilies ecologically similar.	Wiggins 2004 (pupal cocoon); Graf et al. 2008 and Cummins et al. 2019 (larval habitat).
Dipseudopsidae	Lotic-depositional.	2 (black)	Open cocoon in buried larval sand tube.	2 (green)	Subfamilies Dipseudopsinae and Hyalopsychinae (the only subfamilies) (Ross and Gibbs 1973).	Ulmer 1957; Marlier 1962; Gibbs 1968 (Dipseudopsinae: *Dipseudopsis*, *Protodipseudopsis*); Wallace et al. 1976 (Hyalopsychinae: *Phylocentropus*).
Pseudoneureclipsidae	Lotic-depositional.	2 (black)	Open cocoon in new or larval sand tube on substrate surface.	2 (green)	*Pseudoneureclipsis* and *Antillopsyche* (the only genera).	Gibbs 1973; Tachet et al. 2001 (*Pseudoneureclipsis*).
Xiphocentronidae	Lotic-depositional, including above water surface (hygropetric?).	2 (black)	Open cocoon in larval sand tube, possibly hygropetric--Sturm 1960.	2 (green)	Proxiphocentroninae: *Proxiphocentron*, larva unknown (Schmid 1982); Xiphocentroninae: *Drepanocentron* (Vilarino et al. 2022).	Genco et al. 2020 (*Drepanocentron*).
Psychomyiidae	Lotic.	1 (green)	Open cocoon in new, long-dome shelter.	2 (green)	(Psychomyiinae: *Metalype*), (Tinodinae: *Lype*) (Li and Morse 1997)	Edington and Hildrew 1995 (*Metalype*, *Lype*).
Ecnomidae	Lotic-depositional, lentic.	2 (black)	Open or closed cocoon in new, long-dome shelter.	2 (green)	*Daternomina*, *Parecnomina* (Johanson and Espeland 2010).	Cartwright 1997 (*Daternomina*); Gibbs 1973 (*Parecnomina*).
Polycentropodidae	Lotic-depositional.	2 (black)	Open cocoon in new, long-dome shelter.	2 (green)	*Neureclipsis* (Johanson et al. 2012).	Edington and Hildrew 1995; Inaba et al. 2014; Wiggins 1996 (*Neureclipsis*).

^1^ Data mainly from Wiggins 2004.

**Table 3.** Ancestral traits for habits of larvae of basal taxa of Lepidoptera (Kawahara et al. 2019) and the Trichoptera families for which phylogenetic relationships were inferred by Ge et al. (2024a), with Ptilocolepidae from Frandsen et al. (2024), showing color codes for inferred ancestral nodes in Fig. 3.

Taxon	Habit (Trait 5)	Codes for Trait 5	Reference(s) for Inferred Basal Lineage(s) of Terminal Taxa	Reference(s) for Traits of Basal Lineage(s)
Angiospermavora	Terrestrial sprawlers	4 (yellow)	Mitter et al. 2017, Kawahara et al. 2019.	Stehr 1987 (Angiospermavora families).
Heterobathmiidae	Burrower (miner in *Nothfagus* leaves)	1 (brown)	Mitter et al. 2017, Kawahara et al. 2019.	Kristensen and Nielsen 1983.
Agathiphagidae	Burrower (in Kauri seeds)	1 (brown)	Mitter et al. 2017, Kawahara et al. 2019.	Dumbleton 1952.
Micropterigidae	Semi-aquatic sprawlers	3 (black)	Mitter et al. 2017, Kawahara et al. 2019.	Davis and Landry 2012.
Ptilocolepidae	Semi-aquatic sprawlers	3 (black)	*Palaeagapetus* and *Ptilocolepus* (the only genera).	Ito et al. 2014; Waringer and Graf 2002 .
Hydroptilidae	Submerged sprawlers	2 (green)	*Stactobia* (Marshall 1979).	Graf et al. 2008; Waringer and Graf 2011.
Glossosomatidae	Clingers	0 (white)	*Anagapetus* and *Glossosoma* (Robertson and Holzenthal 2013).	Wiggins 1996; Cummins et al. 2019.
Hydrobiosidae	Clingers	0 (white)	*Apsilochorema* (Ward et al. 2004).	Holzenthal et al. 2007.
Rhyacophilidae	Clingers	0 (white)	*Fansipangana* (larva unknown) and *Rhyacophila* (McLaughlin et al. 2019).	Graf et al. 2008.
Phryganopsychidae	Submerged sprawlers	2 (green)	*Phryganopsyche* (only genus).	Wiggins 2004.
Phryganeidae	Clingers, sprawlers	0 (white)	*Yphria* (Wiggins 1998).	Wiggins 1998.
Brachycentridae	Clingers	0 (white)	*Eobrachycentrus* (Flint 1984).	Wiggins 1965, 1996.
Lepidostomatidae	Climbers, sprawlers, clingers	0 (white)	[Basal lineages of Lepidostomatinae and Theliopsychinae have not been inferred.]	Wiggins 2004; Cummins et al. 2019.
Uenoidae	Clingers	0 (white)	*Sericostriata* (Wiggins et al. 1985)	Wiggins et al. 1985.
Goeridae	Clingers	0 (white)	[Basal lineages of Goerinae, Larcasinae, and Lepaniinae have not been inferred. Traits are almost universal in the family.]	Wiggins 1996, 2004.
Apataniidae	Clingers	0 (white)	*Apataniana* (Ge et al. 2024b).	Waringer and Malicky 2016.
Limnephilidae	Clingers	0 (white)	*Ecclisomyia*, *Philocasca* (Vshivkova et al. 2007).	Wiggins 2004; Cummins et al. 2019.
Limnocentropodidae	Clingers	0 (white)	*Limnocentropus* (Wiggins 2004)	Wiggins 2004
Odontoceridae	Burrowers	1 (brown)	Two subfamilies: monotypical Pseudogoerinae and polytypical Odontocerinae, but basal lineages of Odontocerinae have not been inferred. (Wallace and Ross 1971).	Wallace and Ross 1971 (*Pseudogoera*); Wiggins 1996; Holzenthal et al. 2007 (Odontocerinae).
Helicopsychidae	Clingers	0 (white)	*Rakiura* (Johanson 1998; Johanson et al. 2017).	Traits of *Rakiura* not described, apparently as for *Helicopsyche* (Johanson 1998).
Sericostomatidae	Sprawlers	2 (green)	Larvae of basal *Cheimacheramus*, *Rhoizema*, *Petroplax*, and *Notidobiella* unknown; *Gumaga* (Johanson et al. 2017).	Resh et al. 1997.
Calamoceratidae	Sprawlers	2 (green)	Two subfamilies: Monotypical Anisocentropinae and polytypical Calamoceratinae, but basal lineages of Calamoceratinae have not been inferred (Prather 2002).	Wallace and Sherberger 1970 (*Anisocentropus*).
Molannidae	Sprawlers	2 (green)	*Molanna* and *Mollanodes* (the only genera).	Graf et al. 2008; Cummins et al. 2019.
Leptoceridae	Climbers, sprawlers, clingers, swimmers	0 (white)	*Grumichella* and *Leptorussa* (Malm and Johanson 2011); *Russobex* (StClair 1988).	Habits of *Grumichella*, *Leptorussa*, and *Russobex* unknown.
Hydropsychidae	Clingers	0 (white)	Arctopsychinae: *Arctopsyche*, *Parapsyche* (Geraci et al. 2005).	Cummins et al. 2019; Graf et al. 2008.
Stenopsychidae	Clingers	0 (white)	*Pseudostenopsyche* and *Stenopsychodes* larvae unknown; *Stenopsyche* is the only other genus in the family.	Tanida 2002.
Philopotamidae	Clingers	0 (white)	Larvae of basal subfamily Rossodinae (*Rossodes*) are unknown--Gibon 2013; Blahnik 2005; larvae of both remaining subfamilies ecologically similar.	Graf et al. 2008; Cummins et al. 2019.
Dipseudopsidae	Burrowers (miners in sand)	1 (brown)	Subfamilies Dipseudopsinae and Hyalopsychinae (the only subfamilies) (Ross and Gibbs 1973).	Ulmer 1957; Marlier 1962; Gibbs 1968 (Dipseudopsinae: *Dipseudopsis*, *Protodipseudopsis*); Wallace et al. 1976 (Hyalopsychinae: *Phylocentropus*).
Pseudoneureclipsidae	Clingers	0 (white)	*Pseudoneureclipsis* and *Antillopsyche* (the only genera).	Gibbs 1973; Tachet et al. 2001 (*Pseudoneureclipsis*).
Xiphocentronidae	Clingers	0 (white)	Proxiphocentroninae: *Proxiphocentron*, larva unknown (Schmid 1982); Xiphocentroninae: *Drepanocentron* (Vilarino et al. 2022).	Genco et al. 2020 (*Drepanocentron*).
Psychomyiidae	Clingers	0 (white)	Psychomyiinae: *Metalype*; Tinodinae: *Lype* (Li and Morse 1997).	Edington and Hildrew 1995 (*Metalype*, *Lype*).
Ecnomidae	Clingers	0 (white)	*Daternomina*, *Parecnomina* (Johanson and Espeland 2010)	Cartwright 1997 (*Daternomina*); Gibbs 1973 (*Parecnomina*)
Polycentropodidae	Clingers	0 (white)	*Neureclipsis* (Johanson et al. 2012)	Edington and Hildrew 1995; Inaba et al. 2014; Wiggins 1996 (*Neureclipsis*)

**Figure 1.**
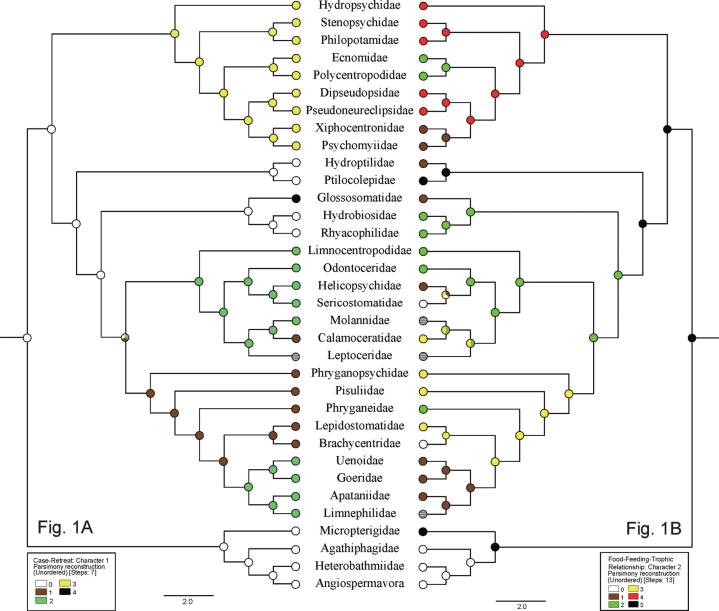
Evolution of case/retreat traits and food traits for Trichoptera and basal Lepidoptera families on phylogeny from Ge et al. (2024a), with modifications from Frandsen et al. (2024) and Kawahara et al. (2019). A. Evolution of case-retreat traits (Character 1), with code 0 (white) = free-living or without case or retreat, 1 (brown) = portable plant-tube case, 2 (green) = portable mineral-tube case, 3 (yellow) = fixed silk retreat, 4 (black) = portable tortoise case, and 5 (stripe) = unknown trait; B. Evolution of food-feeding-tropic relationships traits (Character 2), with code 0 (white) = shredding herbivores-vascular plants, 1 (brown) = grazers and gatherers, 2 (green) = predators, 3 (yellow) = shredding detritivores, 4 (red) = collecting filterers, 5 (black) = shredding herbivores-bryophytes, and 6 (stripe) = unknown trait.

The Case/Retreat category (Table 1) was segregated into five groups of traits for the families: free-living (code 0, without case or retreat), portable plant-tube case (1), portable mineral-tube case (2), fixed silk retreat (3), or portable tortoise case (4). These traits are color-coded white, brown, green, yellow, and black, respectively, in Fig. 1A. Because early instars of Ptilocolepidae and Hydroptilidae live freely and because of the biogenetic law, we included those families in the free-living group.

The Food category (Table 1) was segregated into six groups of traits for the families: shredders-herbivores-vascular plants (code 0), grazers and gatherers (1), predators (2), shredders-detritivores (3), collecters-filterers (4), and shredders-herbivores-bryophytes (5). These traits are color-coded white, brown, green, yellow, red, black, respectively, in Fig. 1B. The food of the ancestral lineages of Limnephilidae is unknown (stripe).

**Figure 2.**
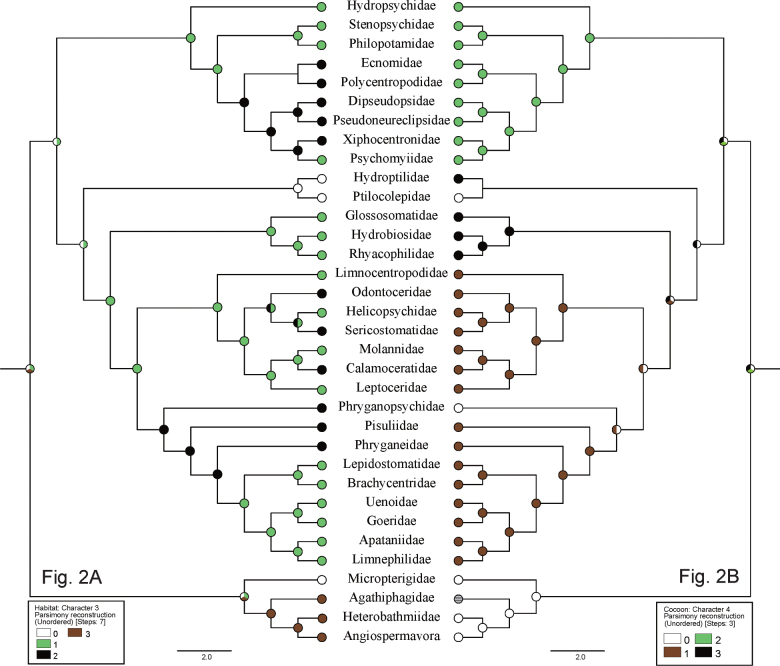
Evolution of habitat traits and cocoon traits for Trichoptera and basal Lepidoptera families on phylogeny from Ge et al. (2024a), with modifications from Frandsen et al. (2024) and Kawahara et al. (2019). A. Evolution of habitat traits (Character 3), with code 0 (white) = madicolous/ hygropetric, 1 (green) = lotic-erosional, 2 (black) = lotic-depositional, 3 (brown) = terrestrial; B. Evolution of cocoon traits (Character 4), with code 0 (white) = closed permeable cocoon, 1 (brown) = open in silk-lined tube case, 2 (green) = open in long-dome shelter or sand tube, 3 (black) = closed semipermeable cocoon, 4 (stripe) = unknown trait.

The Habitat category (Table 2) was segregated into four groups of traits for the families: madicolous/hygropetric (code 0), lotic-erosional (1), lotic-depositional (2), and terrestrial (3). These traits are color-coded white, green, black, and brown, respectively, in Fig. 2A.

The Cocoon category (Table 2) was segregated into four groups of known traits for the families: closed permeable cocoon (code 0); open in silk-lined tube (1); open in long-dome shelter or sand-tube (2); closed semipermeable cocoon (3), sensu Wiggins 2004). These traits are color-coded white, green, black, and red, respectively, in Fig. 2B. The cocoon trait for Agathiphagidae is unknown (stripe).

**Figure 3.**
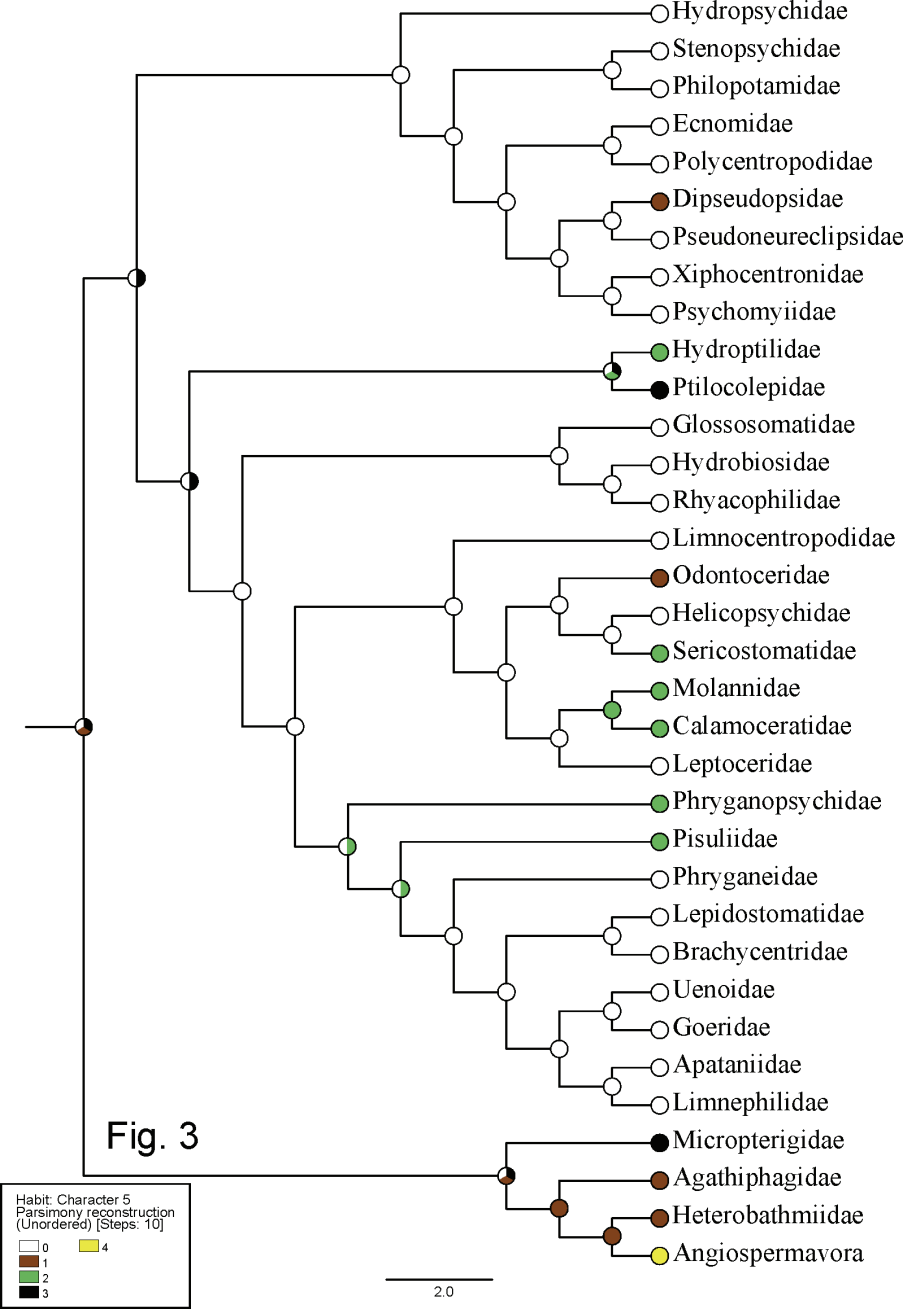
Evolution of habit traits (Character 5) for Trichoptera and basal Lepidoptera families on phylogeny from Ge et al. (2024a), with modifications from Frandsen et al. (2024) and Kawahara et al. (2019). Code 0 (white) = clingers, 1 (brown) = burrowers, 2 (green) = submerged sprawlers, 3 (black) = clingers, 4 (yellow) = terrestrial sprawlers or unknown trait.

The Habit category (Table 3) was segregated into five groups of traits for the families: clingers (code 0), burrowers (1), submerged sprawlers (2), semi-aquatic sprawlers (3), and terrestrial sprawlers (4). These traits are color-coded white, brown, green, black, and yellow, respectively, in Fig. 3.

An Ancestral Character State Reconstruction (ACSR) was conducted for each category of traits. Mesquite v.3.7.0 (http://mesquiteproject.org) was used to perform the ACSR Parsimony Analysis on the encoded family nodes of the phylogeny.

## ﻿Results

The ACSR results are shown at the hypothetical ancestral nodes of Figs 1–3 with the same color notations as for the taxa in Tables 1–3. Regarding larval case or retreat, our analysis supports the conclusion of Frandsen et al. (2024) and Ge et al. (2024a) that the free-living condition, without any type of shelter (e.g., similar to the life-style of early instars of Hydroptilidae and Ptilocolepidae), was probably the ancestral state for Trichoptera (Fig. 1A). Our analysis also supports our hypothesis that caddisfly ancestral larvae were shredding herbivores of bryophytes (Fig. 1B). The ancestral habitat is inconclusive, likely either madicolous/hygropetric or lotic-erosional (Fig. 2A). The type of pupal cocoon was equivocal, as well, either a closed and permeable cocoon or a closed and semipermeable cocoon, or an open silk-lined chamber in a long-dome shelter or sand tube (Fig. 2B). The ancestral larval habit was likely that of a clinger or a semi-aquatic sprawler (Fig. 3).

## ﻿Discussion

### ﻿Trichoptera ancestor and its descendants

Based on their phylogenomic analyses, both Frandsen et al. (2024) and Ge et al. (2024a) agreed that the ancestral larva of Integripalpia in all instars and probably of Trichoptera in at least early instars was “free-living,” i.e., without a portable case or fixed retreat. Now, by comparing the functional traits of the larva of the hypothesized basal lepidopteran lineage (Micropterigidae) and those of modern caddisfly lineages as summarized above, we can infer some additional functional traits of larvae of their Amphiesmenopteran ancestor and the Trichoptera ancestor and of the evolutionary route taken by the Trichoptera ancestor to invade freshwater habitats.

Our analysis concludes that larvae of the free-living caddisfly ancestor probably fed on bryophytes, as those of Micropterigidae and Ptilocolepidae do today. Bryophytes (liverworts, hornworts, mosses) require moist environments for reproduction and are often found living in or immediately beside flowing water and subject to immersion, attached by rhizomes to rocks and other stable substrate on stream edges (Glime 2023). Clinging to or sprawling on semiaquatic bryophytes was probably the common lifestyle of the caddisfly larval ancestor and may have been that of the amphiesmenopteran ancestor as well. The analysis indicated that lotic-erosional or terrestrial habitats are also possibilities. Whatever its habitat may have been, this ancestral caddisfly larva lost its spiracles and either lost or never had a plastron, respiring instead directly through its integument from the cold, oxygen-rich water, apparently making the terrestrial habitat option unlikely. This evolutionary development may also have been possible because of their small size and consequent high surface-to-volume ratio, allowing effective respiration in a freshwater medium. Thus, this ancestral caddisfly larva evolved from a semiterrestrial environment (as in Micropterigidae and Ptilocolepidae) into a permanently submerged lifestyle (as in most Hydroptiloidea and other Trichoptera), radiating to access the many food and habitat resources available under water in the Permian Period.

Precocious production of silk by the active larva, well before time for pupation, apparently evolved for various purposes in the ancestral Trichoptera larva, whether for retreat building (as in modern Annulipalpia) or larval case construction (as in most modern Integripalpia). The primitive caddisfly larva also constructed, apparently for the first time in Amphiesmenoptera, a dome-like pupation shelter of silk and substrate materials, an evolutionary development or synapomorphy of Trichoptera that is observed in basal lineages of both Annulipalpia and Integripalpia. The larva then spun a closed permeable or semipermeable cocoon inside this shelter, fusing the cocoon to its inner walls (Hydroptilidae) or leaving it mostly free (other basal lineages of modern Integripalpia), or the larva spun an open or closed cocoon as in modern Annulipalpia. In Integripalpia, larvae of the tube-case-making Phryganides pupated in their tube cases after lining them with silk and constructing tough silken mesh at either end, then making undulating ventilation movements to propel the unidirectional flow of water through the tube (Wiggins 2004).

### ﻿Integripalpia ancestor and its descendants

Considering the recently published phylogenetic hypotheses (Thomas et al. 2020; Frandsen et al. 2024; Ge et al. 2024a), semipermeable cocoons may have evolved independently in Hydroptilidae and the Glossosomatidae + Rhyacophiloidea lineage; alternatively, semipermeable cocoons may have first appeared in the Integripalian ancestor and been abandoned in the Ptilocolepidae lineage and Phryganides. A further development in the Integripalpia lineage was the precocious construction of the pupal shelter in early instars (immediately on molting into the hypogastric last instar of Hydroptiloidea and in all instars of Glossosomatidae). Wiggins (2004) suggested that the hydroptiloid purse case and glossosomatid tortoise case are homologous because they are both based on two sheets of case materials fastened together at lateral seams; if these case-construction behaviors are homologous, the precocious construction of a pupal shelter apparently was abandoned in Rhyacophiloidea to facilitate rapid movement for capturing prey, delaying shelter construction until immediately before pupation; if they are not homologous, as Frandsen et al. (2024) concluded, the larval ancestor of Glossosomatidae+Rhyacophiloidea apparently was free-living, with the Glossosomatidae ancestor evolving tortoise case construction behavior independently.

The portable tubular cases of Phryganides larvae do not appear to be homologous with the portable cases of Hydroptiloidea and/or Glossosomatidae (Wiggins 2004). Nevertheless, this tubular type of case may also, like those of hydroptiloids and glossosomatids, have evolved as a precocious pupation shelter, initially for larval protection. For pupation, the tubular case was retained and modified by the production of silken sieve plates at the vulnerable ends, or the larval case was abandoned and a new protective tube shelter was constructed as in *Phryganopsyche* (Phryganopsychidae), *Yphria* (Phryganeidae), and *Ecclisomyia* (Limnephilidae) (Wiggins 2004; Givens 2018). Besides its value as camouflage and physical protection, a selective advantage of the tube was for improved respiration by larval and pupal undulations inside the tube, continuously directing a fresh supply of oxygenated water over the body. This adaptation allowed various lineages of Phryganides to invade quiet-water habitats with lower concentrations of dissolved oxygen (Mackay and Wiggins 1979). Most case-making larvae of basal Integripalpia included minerals of various sizes or filamentous algae into their larval/pupal cases. The incorporation of angiosperm leaf pieces in some later lineages, especially in earlier lineages of infraorder Plenitentoria, became possible after the evolution of that major plant clade (Morse et al. 2019).

### ﻿Annulipalpia ancestor and its descendants

Like the larvae of Rhyacophiloidea and early instar larvae of Hydroptiloidea, the larvae of most Annulipalpia have a campodeiform shape. They also evolved strong anal prolegs and tarsal claws for maneuvering efficiently on the substrate. Hydropsychidae are presently thought to be the basal lineage of Annulipalpia (Frandsen et al. 2024; Ge et al. 2024a) and species of their subfamily Arctopsychinae are considered the descendants of the basal lineage of Hydropsychidae (Geraci et al. 2005). Modern species of Arctopsychinae characteristically live in some of the fastest flowing mountain-stream habitats. The functional changes that allowed a free-living, wet-habitat ancestral caddisfly larva to invade lotic-erosional ecotones characteristically occupied by modern arctopsychines are unclear and must have been dramatic, a scenario inviting future investigation.

## ﻿Conclusions

An appreciation of the importance of functional traits for understanding freshwater communities and their necessary biotic and abiotic characteristics has increased in recent years (Graf et al. 2008; Merritt et al. 2019). Inference of ancestral functional traits helps put modern traits in their historical context and provides a foundation for predicting and confirming yet-unknown traits still being investigated for the modern fauna (Morse and White 1979).

## ﻿Additional information

### Use of AI

No tools based on artificial intelligence technologies were used in the conduct of this research or in the preparation or editing of the resulting manuscript.

### Author ORCIDs

Xinyu Ge https://orcid.org/0000-0001-7375-504X

John C. Morse https://orcid.org/0000-0003-3187-4045

### Data availability

All of the data that support the findings of this study are available in the main text.
